# German Medical Officers for Prisoners' Camps?

**Published:** 1917-08-04

**Authors:** J. King

**Affiliations:** House of Commons


### THE EDITOR'S LETTER-BOX.

### German Medical Officers for Prisoners' Camps?

*To the Editor of* The Hospital.

Sir,—I have endeavoured to raise in Parliament, without much effect so far, the waste of British medical men, kept on work in the large camps where German and other enemy aliens (civilians) are interned. In one of these (Isle of Man) there are eight British doctors whose whole time is so employed. Add to these the medical attendances required by the thousands of prisoners of war in camps, settlements, and on special work in the United Kingdom, and you have an immense field for the many German civilian doctors, interned or prisoners, but not medically employed. I am told that it was tried (long ago?) with "not very satisfactory results" (why?) to employ the captive doctors. Is there not a possibility of employing German doctors who are there? Cannot you or some of your readers take up this matter?—Yours truly,

J. King.

House of Commons, July 30, 1917.

